# Size-Exclusion Chromatography-based isolation minimally alters Extracellular Vesicles’ characteristics compared to precipitating agents

**DOI:** 10.1038/srep33641

**Published:** 2016-09-19

**Authors:** Ana Gámez-Valero, Marta Monguió-Tortajada, Laura Carreras-Planella, Marcel·la Franquesa, Katrin Beyer, Francesc E. Borràs

**Affiliations:** 1 REMAR-IVECAT Group, Health Science Research Institute Germans Trias i Pujol, Can Ruti Campus, Badalona, Spain; 2 Department of Pathology, Hospital Universitari and Health Sciences Research Institute Germans Trias i Pujol, Universitat Autònoma de Barcelona, Spain; 3 Nephrology Service, Germans Trias i Pujol University Hospital, Badalona, Spain

## Abstract

Extracellular vesicles (EVs) have become an attractive field among the scientific community. Yet, a major challenge is to define a consensus method for EVs isolation. Ultracentrifugation has been the most widely used methodology but rapid methods, including Size Exclusion Chromatography (SEC) and/or precipitating agents such as Polyethylene glycol (PEG) or PRotein Organic Solvent PRecipitation (PROSPR) have emerged. To evaluate the impact of these different methods on the resulting EV preparations, plasma EVs were isolated using SEC, PEG and PROSPR, and their total protein content, NTA and Cryo-electron microscopy profiles, and EV-markers were compared. Also, their effect on recipient cells was tested. Low protein content and Cryo-EM analysis showed that SEC removed most of the overabundant soluble plasma proteins, which were not removed using PEG and partially by PROSPR. Moreover, only SEC allowed the detection of the EV-markers CD9, CD63 and CD81, LGALS3BP and CD5L, suggesting a putative interference of the precipitating agents in the structure/composition of the EVs. Furthermore, PEG and PROSPR-based EV isolation resulted in reduced cell viability *in vitro*. These results stress that appropriate EV-isolation method should be considered depending on the forthcoming application of the purified EVs.

The vast majority of eukaryotic cells release extracellular vesicles (EVs), which are involved in cell-cell communication and also pathogenic processes^1^. EVs have also been envisaged as drug and gene delivery vectors for cell-free therapeutic approaches^2^,^3^. In addition, due to their particular composition and their presence in several body fluids such as blood, urine or saliva among others^4^,^5^,^6^, EVs have importantly emerged as potential reservoirs of biomarkers for many pathological processes and patients’ follow-up^7^,^8^. One of the major drawbacks in the development of EVs as potential biomarkers is their sometimes time-consuming isolation procedures, which involve density gradients and differential ultracentrifugation^9^. These techniques cannot be easily adapted to the clinical labs, boosting the development of alternative methodologies and approaches for EVs isolation^10^.

In this sense, new methods based on aggregating agents such as polyethylene glycol (PEG), or size-exclusion and immunoaffinity columns have been described^11^,^12^,^13^,^14^,^15^. More recently, PRotein Organic Solvent PRecipitation (PROSPR) has been reported as a new feasible EV-purification protocol based in protein precipitation by cold acetone^16^. Although most of these methods meet the expectations of quickly obtaining EVs for biomarker determination in a clinical setting, the final composition of the “isolated” EVs, the abundance of contaminating proteins and the effect of the isolation technique on the EVs is still a matter of controversy. In this study, we aimed to characterize the EVs isolated from plasma and cell culture samples using three different methods, Size Exclusion Chromatography (SEC), PEG and PROSPR, to compare how each method influences the obtained EV preparation. Our results suggest that, although vesicles could be isolated by PROSPR and SEC protocols, only SEC seems not to alter the size and the vesicular characteristics.

## Results and Discussion

### Protein content from isolated EVs

**Figure 1:**
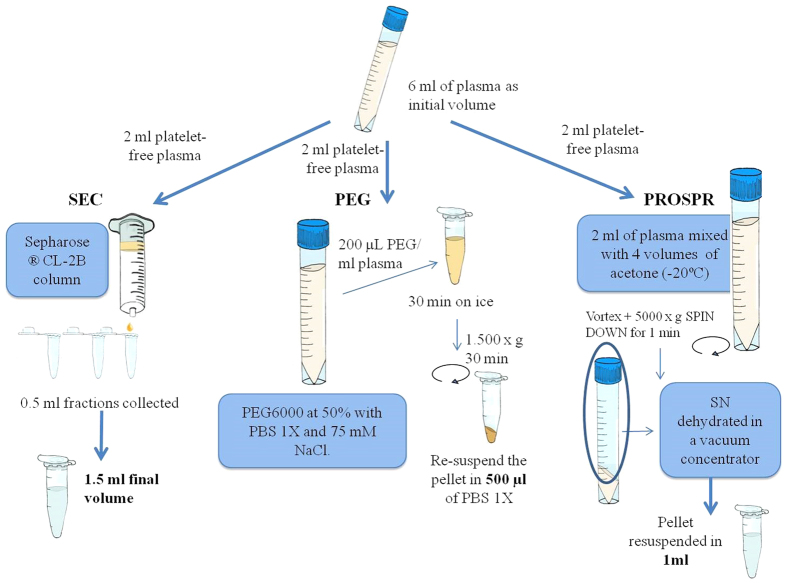
Schematic representation and description of the main steps involved in the three methodologies used for the isolation of EVs.

**Figure 2:**
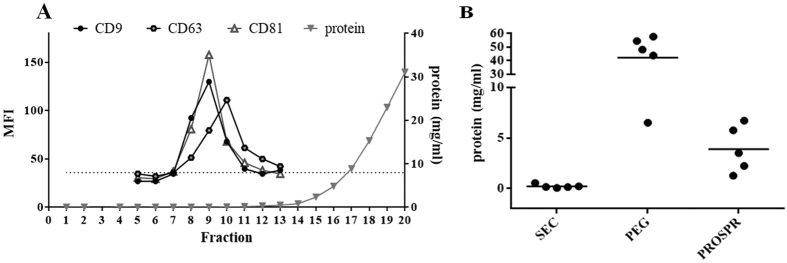
Protein content of EVs is dependent of the isolation method. (**A**) Representative experiment of plasma-derived EVs eluted in low protein fractions after SEC isolation. A total of 20 fractions of 0.5 mL were collected and the presence of tetraspanins CD9, CD63, CD81 and total protein content were determined. The three top MFI tetraspanin fractions of each experiment were pooled for further analyses. A representative sample of five independent experiments is shown. The isotype negative control is represented as a dashed line. (**B**) Protein concentration from different EV-enriched preparations measured by absorbance at 280 nm and calculated using a BSA standard curve. Protein content from SEC-EVs was determined in the pooled fractions. Five independent experiments are shown.

The starting volume of platelet-free plasma used for each isolation method was 2 mL. From this initial volume, precipitation of EVs using PROSPR and PEG rendered a final volume of 1 mL and 500 μL, respectively (Fig. 1). Using SEC, EVs from the initial 2 mL of platelet-free plasma were finally distributed in several tetraspanin-positive fractions of 500-μl (Figs 1 and 2A), which were pooled in a single tube of 1.5 mL volume.

First, the total protein of the obtained EV preparations was estimated by measuring the absorbance at 280 nm in five different independent samples. While PROSPR samples rendered a mean of 3.9 mg of total protein (3.9 ± 1.1 mg, n = 5), PEG-precipitation resulted in 5 times increased protein content (21.1 ± 10.4 mg, n = 5) (Fig. 2B). Conversely, protein content was undetectable in SEC-pooled EV fractions (0.3 ± 0.3 mg, n = 5), as most of the soluble proteins eluted in later fractions (fraction 15 onwards in Fig. 2A), devoid of EVs as revealed by the absence of tetraspanin markers. Thus, high quantities of soluble plasmatic proteins are pelleted along with EVs when using precipitating methods. Therefore, attention should be paid when these EV-enriched samples are subjected to proteomic analysis as EV-related proteins might be masked by over-represented plasma proteins. While a huge part of these soluble contaminants are not to be co-precipitated by cold acetone precipitation, as revealed by a lower protein content, the undetectable amount of total protein still point to SEC as the cleanest isolation method removing most of the overabundant plasma proteins.

### EV characterization by flow cytometry and western blot

**Figure 3:**
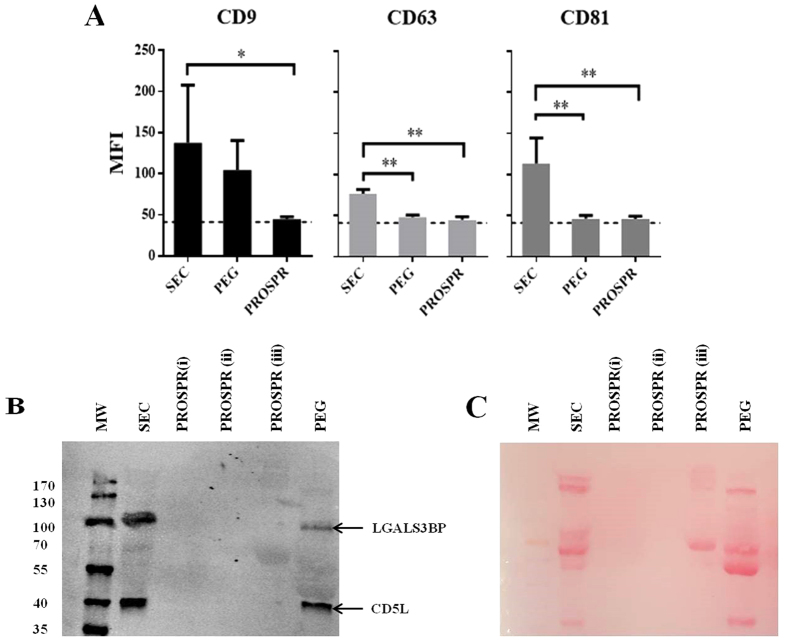
EV markers detection by flow cytometry and Western blot analysis. (**A**) MFI values of the tetraspanins CD9, CD63 and CD81 in the SEC-pooled fractions, PEG precipitate and PROSPR product by bead-based flow cytometry. Bars represent mean+/−SD of four independent experiments. Differences with *P < 0.05, and **P < 0.005, were considered statistically significant by One-way ANOVA, Tukey test. (**B**) Western blot analyses of CD5L and LGALS3BP proteins from SEC isolated EVs, PEG and PROSPR products. SEC-EVs were concentrated using a 100 kDa-Amicon Ultra and dehydrated in a vacuum concentrator. Three different PROSPR concentrating preparations were loaded. First, the supernatant from PROSPR protein precipitation was dehydrated in a vacuum concentrator (miVac Quattro) at room temperature, and resuspended in 1 mL as originally reported (i). To further concentrate the sample, (i) was then ultrafiltered as in SEC samples and dehydrated to 30 μL of final volume (ii). Alternatively, the 5000× g supernatant was directly concentrated using the 100 kDa-Amicon Ultra (Millipore), reaching a final volume of 100 μL (iii). (**C**) Ponceau S Staining of the blotting membrane.

The presence of some classical exosomal markers -tetraspanins CD9, CD63 and CD81- was determined by bead-based flow cytometry and used to identify fractions containing EVs in SEC isolation (Fig. 2A). The presence of all the three tetraspanins was clearly confirmed on SEC pooled EV-enriched fractions (Fig. 3A). However, when tetraspanins were evaluated in PEG and PROSPR preparations from the same samples, CD63 and CD81 were undetectable. Only CD9 could be determined in samples obtained by PEG but not when using PROSPR (Fig. 3A). Of note, Gallart -Palau *et al.*^16^ reported the presence of tetraspanins CD9 and CD63 in PROSPR-EVs samples by western blot. Nevertheless, in our flow cytometry analyses performed using non-altered EVs the presence of these tetraspanines could be detected, thus facilitating their determination. Regarding PEG-isolation, it is possible that some epitopes for CD63 and CD81 could be altered by the isolation method, or masked by other contaminant proteins present in the sample.

To further extend the analyses of EV samples, the presence of LGALS3BP and CD5L was also determined using western blot. These markers have been previously described in several proteomic analyses of plasma-derived EVs^13^. While both LGALS3BP and CD5L were detected in SEC and PEG preparations, surprisingly no expression was found in PROSPR-enriched samples (Fig. 3B). The membrane was also stained with Ponceau S Solution to visualize the purity of the different loaded samples (Fig. 3C). Despite similar amount of total protein (based on absorbance at 280 nm) were loaded in each case, an unexpected result was that only in ultrafiltered PROSPR-vesicles some stained protein was detected in the membrane. Nevertheless, the PROSPR samples were negative for both widely accepted EV markers. These results suggest that SEC-EV isolation minimally affect EV composition and permits the detection of EV markers using different methods.

### EV characterization by NTA and Cryo-EM

**Figure 4:**
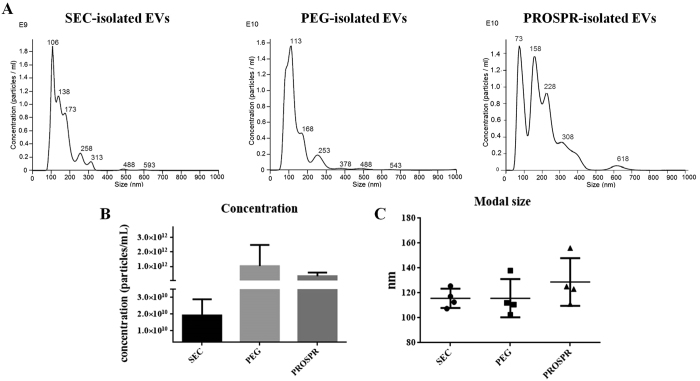
EVs characterization by Nanoparticle Tracking Analysis (NTA). (**A**) Representative size distribution profiles of EVs isolated by SEC, PEG or PROSPR precipitation. (**B**) Concentration (particles/mL) and (**C**) modal size (nm) of EV samples according to NTA measures. The median +/−SD is reported for four independent experiments from four individual samples carried out in parallel with each method.

To further characterize the isolated EVs obtained from each isolation method, concentration and size distribution of EVs were determined in SEC-pooled EVs and PROSPR and PEG preparations by Nanoparticle Tracking Analysis (NTA) (Fig. 4). SEC-purified EVs yielded 1.9E + 10 particles/mL with an average modal size of 116.4 ± 7.7 nm (median ± SD, n = 4). PROSPR purification clearly increased the concentration (3.3E + 11 particles/mL), although showed a slightly higher particle size (136.0 ± 18.4 nm, n = 4). Finally, PEG-isolated EVs resulted in a 2-log increase of particles compared to SEC (1.0E + 12 particles/mL) with equivalent size (116.0 ± 15.4 nm n = 4). The NTA profiles also showed a major peak of abundant vesicles of around 110 nm to 120 nm for SEC and PEG preparations, whilst PROSPR showed several peaks, some of them well over 200 nm (Fig. 4A).

**Figure 5:**
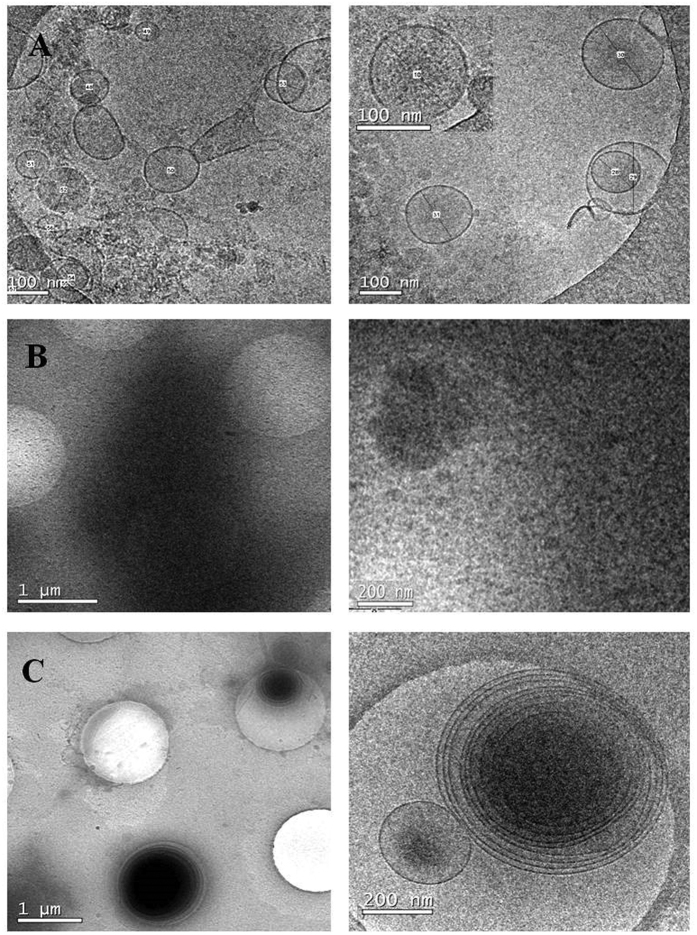
Cryo-EM images. (**A**) Pooled SEC fractions showed single vesicles of size between 80–200 nm. (**B**) No vesicles were found in Cryo-EM images from PEG precipitated samples. Electron dense particles covered the whole grid indicating co-precipitation of contaminants. (**C**) PROSPR isolated vesicles were visualized as concentric multi-layered vesicles, mostly measuring from 300 to 500 nm. Scale bars are shown for each image.

**Figure 6:**
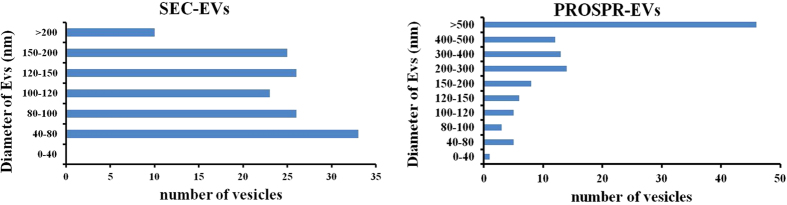
Distribution of the diameter (nm) of vesicles found in SEC and PROSPR preparations. Vesicles detected in Cryo-EM images from either SEC (143 vesicles) or PROSPR (113 vesicles) preparations were counted and the diameter measured using Image J Program. PEG isolated EVs were not detected in Cryo-EM images.

To better delineate EV characteristics, isolated vesicles were analysed using cryo-EM. Microscopy images revealed the presence of 80–200 nm sized vesicles in the SEC-pooled fractions (Fig. 5A). Images also showed very few contaminant particles in these preparations. Conversely, we were not able to visualize any vesicles in the PEG-isolation preparation, but some dense contaminant aggregates (Fig. 5B). As for EVs obtained by PROSPR, an unexpected observation was that most of them appeared merged in concentric multi-layer vesicles (Fig. 5C), which could explain the increased size found in NTA experiments. The compilation of EV sizes gathered from several cryo-EM images also confirmed the results of the NTA analysis (Fig. 6). Given the cryo-EM profile of the PROSPR isolated vesicles, it is tempting to speculate that specific antibodies did not detect tetraspanins by flow cytometry in the native structure of the vesicles due to EV fusion. Our current results cannot clarify whether PROSPR-EVs have fused or are artificially multilayered during the Cryo-microscopy preparation. Some studies described a “degradative” potential of organic solvents such as acetone, which could increase the membrane fluidity and therefore fusion^17^. Comparatively, multilayering is scarce in cryo-microscopy preparations from SEC-EVs, thus indicating that the observed pattern may not be attributed to random distribution.

Cryo-EM results indicate that SEC yields individual, clean EVs while PEG seems to co-precipitate proteins and contaminants, whilst cold acetone precipitation could induce vesicular fusion, which may affect the membrane and properties of the isolated vesicles. Considering all the observations, the putative interference of the isolation methods in the structure and/or composition of the vesicles should be taken into account depending on their forthcoming application.

### Effect of isolated EVs on *in vitro* cultured cells

The variable results in protein content, vesicular size and detection of tetraspanins of the different EV preparations could also suggest modifications in the vesicular properties that may affect their function. A preliminary approach to evaluate the effect of the different isolation protocols on EV’s function was to observe their impact in the survival of different cells when added *in vitro*. Using the human monocytic cell line THP-1 as a source of EVs, vesicles were isolated from cell culture supernatants (10^6^ EV producing cells) using the three different methodologies. Then, 10^5^ target cells were cultured in the presence of EVs and cell viability was evaluated at different time points.

**Figure 7:**
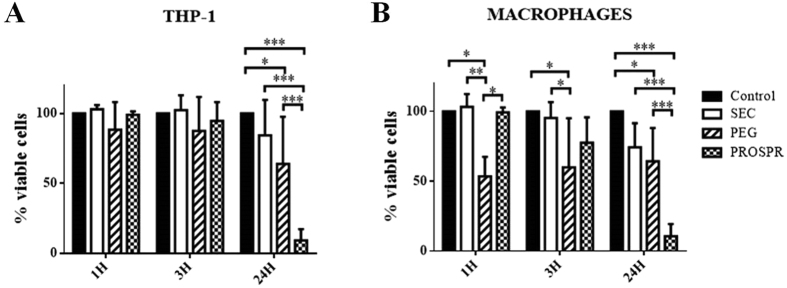
EV effect on cell viability. (**A**) Viability of THP-1 cells and (**B**) THP-1-derived macrophages incubated with THP-1-EVs from SEC, PEG or PROSPR isolation. The results are expressed as the percentage of viable cells relative to the control condition (PBS) in each particular experiment. Bars represent mean+/−SD of 3 to 5 independent experiments. Differences with *P < 0.05, **P < 0.005, and ***P < 0.0001 were considered statistically significant by Two-way ANOVA, Tukey test.

Target cells included the THP-1 cell line itself and THP-1-derived macrophages. In all the conditions tested, cell viability was faintly affected up to 3 hours when EVs derived from SEC and PROSPR were added (Fig. 7). In contrast, THP-1-derived macrophages exposed to PEG-isolated vesicles showed a clearly reduced viability after 1 and 3 hours at 37 °C (Fig. 7B). When cells were analyzed at longer time points (24 h), the reduced cell viability observed in PEG-EV treated cultures did not show further reduction, while THP-1 and also macrophages dramatically decreased viability when exposed to PROSPR-derived vesicles (Fig. 7 and Supplemental Fig. 1, panels Di,ii). Noticeably, cultures with SEC-EVs had similar behaviour compared to control conditions in all time points tested. These results clearly suggest that some of the methods used to isolate EVs may affect cell viability, an additional aspect to be considered when choosing the EV isolation to be used in functional assays and possible clinical applications.

To summarize, the results of this study show that EV isolation methodologies differently affect the vesicle preparations and alter EV profiles (summarized in Table 1). In this line, although precipitating agents could represent a quick and easy method suitable for implementation in the clinics, our results suggest that they could hamper EV-associated proteome discovery and be especially detrimental for therapeutic applications of EVs. In this sense, a recent study has referred to the influence of isolation methods in the biological activity of EVs, as they probably affect EV-membrane interactions^18^. The toxicity of PEG- and PROSPR-derived EV preparations observed in some cell lines should receive special attention when using these vesicles in clinical applications for EV-based therapies. Finally, our results suggest not only that SEC is capable of removing most of the abundant proteins contained in a body fluid but also it maintains the vesicular structure and conformation, thus making this procedure ideal for biomarker discovery as well as for therapeutic applications. The main disadvantage of this technique could be the limited quantity of EVs recovered, although the scale-up of SEC is possible and encourages its use for the EV-isolation for therapeutic uses.

## Materials and Methods

### Sample collection and Ethic Statement

Plasma samples were obtained from healthy donors at the Germans Trias i Pujol Hospital of Badalona (Spain). Informed consent was obtained from all subjects and the study was approved by the “Germans Trias i Pujol” Ethical Committee according to the Declaration of Helsinki (BMJ 1991; 302:1994). All samples were analyzed anonymously and all the methods were carried out in accordance with the relevant guidelines.

Peripheral blood was collected following standard procedures that minimize contamination by platelet and platelet-derived vesicles^19^,^20^,^21^. Briefly, by venous puncture, the first millilitre of blood was discarded before collection of 3 mL in several trisodium citrate pre-treated tubes (BD, San Jose, CA). After inversion (8–10 times), samples were processed within the 30 minutes of collection. Consecutive centrifugation steps at 400× g for 5 minutes to eliminate red blood cells and 2,500× g for 10 minutes at room temperature to minimize contamination by platelets and platelet-derived vesicles were performed^21^,^22^. Supernatant was subject to a final centrifugation of 13,000× g for 5 minutes to completely obtain a platelet-free plasma sample^23^,^24^. Plasma samples were then processed for vesicle isolation within the same day as represented in Fig. 1.

### EV isolation by Size Exclusion Chromatography (SEC)

Isolation of vesicles by SEC was performed as described by Boïng and colleagues^12^. Succinctly, 12 mL of Sepharose CL-2B (Sigma Aldrich, St. Louis, MO, USA) was stacked in a 20-mL syringe (BD PlasticpakcTM, San Jose, CA) and washed and equilibrated with PBS (Oxoid).

Two mL of plasma were loaded onto the column and fraction collection (0.5 mL per fraction and a total of 20 fractions were collected) started immediately using PBS as elution buffer.

### EV isolation by Polyethylene glycol precipitation (PEG)

The application of polyethylene glycol in EVs purification has recently been reported^25^,^26^. A working solution of PEG was prepared with PEG6000 (Sigma Aldrich) at 50% with PBS and 75 mM NaCl. Two mL of platelet-free plasma were mixed up with 200 μL of 50%-PEG (final concentration of 10%), incubated on ice for 30 minutes and finally centrifuged at 1,500× g for 30 minutes. The obtained pellets were dissolved in 500 μL PBS.

### EVs isolation by PRotein Organic Solvent PRecipitation (PROSPR)

PROSPR procedure was performed as recently published^16^. Briefly, two mL of sample were directly mixed with four times the volume of cold acetone (previously cooled down at −20 °C) (QCA, Química Clínica Aplicada S.A.). After vortexing it, the mixture was centrifuged at 3,000× g for one minute. Extracellular vesicles are expected to be contained in the resultant supernatant, thus this supernatant was dehydrated in a vacuum concentrator (miVac Quattro from Genevac) at room temperature for approximately 1–1.5 hours. Once dried, the resultant EV-containing pellet was resuspended in 500 μL PBS.

### Measurement of Protein content

Protein content was measured by reading absorbance a 280 nm using Nanodrop^®^ ND-1000 from Thermo Scientific. Protein concentration was calculated using a standard curve of bovine serum albumin (BSA) serial dilution also assessed by bicinchoninic acid assay (BCA assay) (Thermofisher Scientific) and Nanodrop.

### Nanoparticle Tracking Analysis

Size distribution and concentration of isolated vesicles were measured in a NanoSight LM10 instrument (Malvern Instruments Ltd, Malvern, UK) equipped with a 638 nm laser and CCD camera (model F-033), and data were analyzed with the Nanoparticle Tracking Analysis (NTA) software (versions 3.1 build 3.1.46). To perform the measurements, detection threshold was set to 5, and blur and Max Jump Distance were set to auto. Samples processed by SEC were diluted 90-100 times with sterile and filtered Phosphate Buffered Saline (PBS) to reduce the number of particles in the field of view below 140/frame. Readings were taken in single capture during 60 s at 30 frames per second (fps), at camera level set to 16 and manual monitoring of temperature.

In the same way, preparations obtained by PROSPR and PEG aggregation were analyzed taking into account the same parameters.

### Bead-based flow cytometry

Fractions from SEC and EV-enriched fractions from PEG and PROSPR were analyzed by flow cytometry to identify the classical exosomal markers CD63, CD9 and CD81. In the same way as previously reported^14^, 50 μL of each fraction were incubated with 0.2 μL aldehyde/sulphate-latex beads (4 μm; Invitrogen, Carlsbad, CA) for 15 min at RT. Once re-suspended in 1 mL bead-coupling buffer (BCB) (PBS supplemented with 0.1% BSA and 0.01% NaN3; Sigma Aldrich), the mix was incubated overnight at RT on rotation. EV-coated beads were then spun down at 2,000× g for 10 min, washed with BCB and centrifuged again at 2,000× g for 10 min. EV-coated beads were then labelled at 4 °C with anti-CD9 (Clone VJ1/20), anti-CD63 (Clone TEA 3/18), and anti-CD81 (clone #G0709, from Santa Cruz Biotech) or polyclonal IgG isotype (Abcam, Cambridge, UK) antibodies for 30 min. After washing with BCB, EV-coated beads were incubated with FITC-conjugated secondary goat anti-mouse antibodies (SouthernBiotech, Birmingham, AL) for 30 min, washed twice with BCB and analyzed by flow cytometry (FacsVerse; BD Biosciences, San Jose, CA) using the FlowJo software (Tree Star, Ashland, OR). A total of 10,000 beads were acquired for each sample and the median fluorescence intensity (MFI) was obtained.

### Western Blot

Samples obtained by the three methodologies were analysed for the presence EV markers by Western Blot. In order to load an equal protein amount into the gel electrophoresis, samples were further diluted or concentrated as needed. The tetraspanin-peak SEC fractions were concentrated using a 100 kDa-Amicon Ultra (Millipore, Merck kGaA, Darmstadt, Germany) and dehydrated in a vacuum concentrator (miVac Quattro, Genevac) at room temperature. PROSPR treated samples were processed in three different ways to concentrate the acetone-rich 5000× g supernatant. First, the procedure described^16^ was strictly followed, dehydrating the supernatant in a vacuum concentrator (miVac Quattro) at room temperature, and resuspended in 1 mL (i). To further concentrate the sample, (i) was then ultrafiltered as in SEC samples and dehydrated to 30 μL of final volume (ii). Alternatively, the 5000× g supernatant was directly concentrated using the 100 kDa-Amicon Ultra (Millipore), reaching a final volume of 100 μL (iii). Finally, PEG samples were diluted in PBS.

Equivalent protein amounts from each sample were mixed with reducing Laemmli Buffer and β-mercaptoethanol (Bio-rad laboratories, USA) and loaded on a Mini-protean TGX Precast Gel from Bio-rad laboratories. After the electrophoresis, proteins were transferred to a low fluorescence PVDF blotting membrane (GE Healthcare, Life Science, Germany) and blocked in 5% non-fat milk in 0.1% Tween-20 PBS for 1 h. Membranes were then incubated with primary antibodies antiCD5L-Biotinylated [1:1000, R&D, BAF2797] and anti-LGALS3BP [1:500 Abcam, Cambridge, UK, 3G8] for 16 h at 4 °C. After three washes in PBS+ 0.1%Tween-20, membranes were incubated with corresponding conjugated secondary antibodies (IRDye-800CW Goat polyclonal anti-mouse IgG H+L and streptavidin protein, DylLight 680 conjugate from ThermoFisher, 21848; both at 1:15000 dilution) for 1 h at room temperature. After three washes in PBS+ 0.1%Tween-20 and two additional washes in PBS, membrane was analysed using an Odyssey Infrared Imager (Li-cor). Total protein loaded in the gel was detected by staining the membrane with Ponceau S Solution for 5 minutes at room temperature.

### Cryo electron microscopy

SEC fractions showing the highest MFI for exosomal markers by bead-based assay in flow cytometry were pooled, the fraction obtained by PEG precipitation and the vesicles isolated using PROSPR were selected (n = 1 for each method) for cryo-TEM microscopy. Vitrified specimens were prepared by placing 3 μl of a sample on a Quantifoil^®^ 1.2/1.3 TEM grid, blotted to a thin film and plunged into liquid ethane-N_2_(l) in the Leica EM CPC cryoworkstation. The grids were transferred to a 626 Gatan cryoholder and maintained at −179 °C. The grids were analyzed with a Jeol JEM 2011 transmission electron microscope operating at an accelerating voltage of 200 kV. Images were recorded on a Gatan Ultrascan 2000 cooled charge-coupled device (CCD) camera with the Digital Micrograph software package (Gatan).

### Cell culture and EV impact

The THP-1 human monocytic cell line was grown in complete medium composed of RPMI1640 (Gibco) supplemented with 10% Fetal Calf Serum (FBS, Gibco), 2 mM L-glutamine (Sigma-Aldrich) 100 U/mL penicillin (Cepa S.L), 100 μg/mL streptomycin (Laboratories Normon S.A.), 1 mM sodium pyruvate and 50 μM β-mercaptoethanol (both from Sigma-Aldrich). Cells were cultured in EV-free complete medium (>16 h 100,000× g ultracentrifuged FBS) with 10 ng/mL phorbol 12-myristate 13-acetate (PMA, Sigma) to promote vesicle production. After incubation for 2 hours, supernatant was collected and EVs were isolated by the SEC, PEG or PROSPR methods as described before. The same amount of cells and derived conditioned medium were processed following each of the three isolation methods.

Macrophages were derived from THP-1 cells by culturing cells with 10 ng/mL PMA for 48 h and further 24 h of resting. For viability assays, 100,000 cells were placed in EV-free complete medium in the presence or absence of EVs. After incubation for 30 min, 1, 3 or 24 hours at 37 °C, cell viability was measured by forward and side scattering and 7AAD (BD Biosciences) staining in a FACSCanto II flow cytometer (BD Biosciences).

### Statistical analysis

Data were tested for statistical significance by one-way or two-way ANOVA followed by Tukey test as indicated in the figure legends using Graph-Pad Prism software. Data were considered statistically significant when p < 0.05.

## Additional Information

**How to cite this article**: Gámez-Valero, A. *et al.* Size-Exclusion Chromatography-based isolation minimally alters Extracellular Vesicles’ characteristics compared to precipitating agents. *Sci. Rep.* **6**, 33641; doi: 10.1038/srep33641 (2016).

## Supplementary Material

Supplementary Information

## Figures and Tables

**Table 1:** Summary of the characterization of EVs isolated using the three different methodologies.

		Size Exclusion Chromatography (SEC)	PolyethyleneGlycol (PEG)	PRotein Organic Solvent PRecipitation (PROSPR)
Initial Volume (mL)		2	2	2
Final Volume (mL)		1.5	0.5	1
Final protein content (mg) of resulting EV preparation		0.3 ± 0.3	21.1 ± 10.4	3.9 ± 1.1
Tetraspanins detection		CD9+/CD63+/CD81+	CD9+/CD63−/CD81−	CD9−/CD63−/CD81−
Other EV markers (Western Blot)		LGALS3BP+/CD5L+	LGALS3BP+/CD5L+	Non detected
Nanoparticle Tracking Analysis	Modal Size (nm)	116.4 ± 7.7	116 ± 15.4	136.0 ± 18.4
	Concentration (particles/mL)	1.9E + 10	1.0E + 12	3.3E + 11
Cryo Electron microscopy		Mostly single vesicles (80–200 nm)	Undetectable	Concentric multi-layer vesicles (>200 nm)
