# Supplementary material for: Size-Exclusion Chromatography-based isolation minimally alters Extracellular Vesicles’ characteristics compared to precipitating agents
Source: Sci Rep. 2016 Sep 19;6:33641. doi: 10.1038/srep33641 (PMC5027519; doi:10.1038/srep33641)
Supplement: Supplementary Information [file srep33641-s1.pdf]

Size-Exclusion Chromatography-based isolation minimally alters Extracellular Vesicles' characteristics compared to precipitating agents

Ana Gámez-Valero[1, 2], Marta Monguió-Tortajada [1], Laura Carreras-Planella [1], Marcel·la Franquesa[1], Katrin Beyer[2], Francesc E. Borràs[1, 3*]

[1]REMAR-IVECAT Group, Health Science Research Institute Germans Trias i Pujol, Can Ruti Campus, Badalona, Spain;
[2]Department of Pathology, Hospital Universitari and Health Sciences Research Institute Germans Trias i Pujol, Universitat Autònoma de Barcelona, Spain;
[3]Nephrology Service, Germans Trias i Pujol University Hospital, Badalona, Spain

Key words: PROSPR, Size Exclusion Chromatography, PEG precipitation, EVs isolation, exosomes

*Correspondence should be addressed to:

F.E.B.: feborras@igtp.cat, Tel: 0034 93 497 86 71; Fax: 0034 93 497 86 68

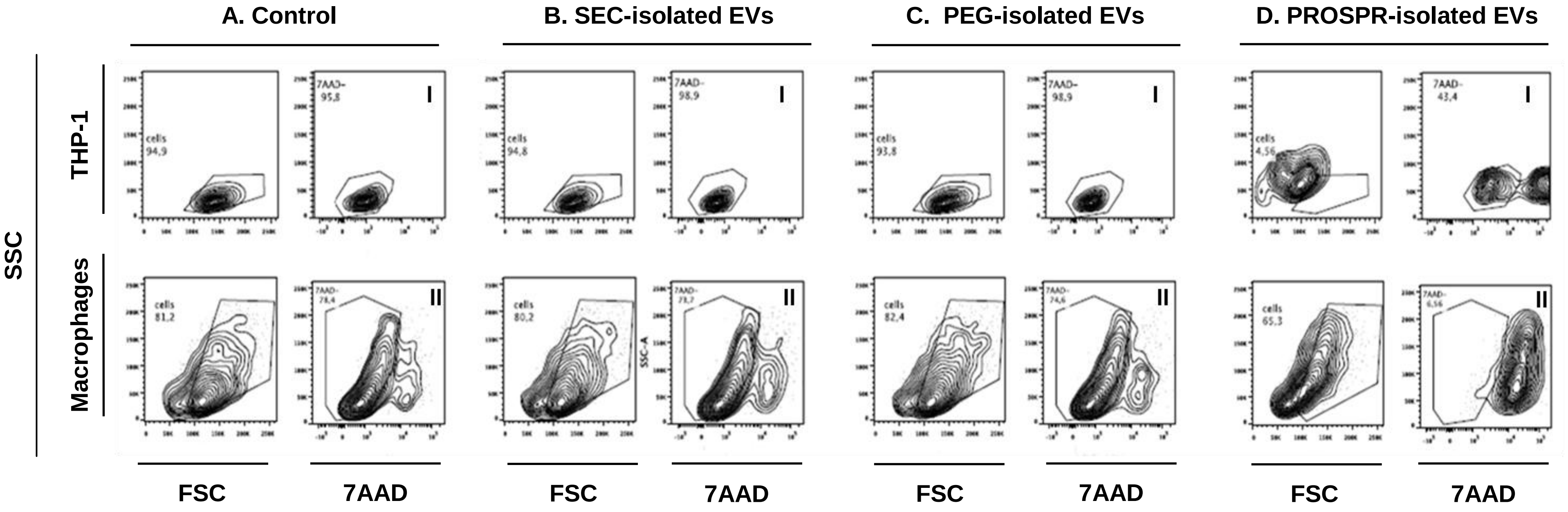

Supplementary Figure 1. Representative flow cytometry plots of cells incubated for 24h at 37ºC with EVs isolated from stimulated THP-1 cells by SEC, PEG or PROSPR methods. Cells exposed to PBS were used as control condition. Viable cells were gated according to their FSC-A/SSC-A profiles and dead cells were gated by 7AAD$^+$ staining. One representative experiment for THP-1 monocytes and for macrophages are shown.
